# The Mental Health Leadership and Advocacy Program (mhLAP): a pioneering response to the neglect of mental health in Anglophone West Africa

**DOI:** 10.1186/1752-4458-8-5

**Published:** 2014-01-27

**Authors:** Jibril Abdulmalik, Woye Fadahunsi, Lola Kola, Emeka Nwefoh, Harry Minas, Julian Eaton, Oye Gureje

**Affiliations:** 1 Department of Psychiatry, University of Ibadan, Ibadan, Nigeria; 2 National Mental Health Consultant, World Health Organization, Nigeria Country Office, Osogbo, Nigeria; 3 Nigeria Co-ordination Office, CBM International, Abuja, Nigeria; 4 Centre for International Mental Health Melbourne; School of Population and Global Health, The University of Melbourne, Melbourne, Australia; 5 CBM Regional Office, Togo, Africa; 6 WHO Collaborating Centre for Research and Training in Mental Health, Neurosciences and Substance Abuse, Department of Psychiatry, University of Ibadan, Ibadan, Nigeria

**Keywords:** Mental health, advocacy, West Africa, Service users, Stakeholders, Stigma, LAMIC

## Abstract

Developing countries in Africa and other regions share a similar profile of insufficient human resources for mental health, poor funding, a high unmet need for services and a low official prioritisation of mental health. This situation is worsened by misconceptions about the causes of mental disorders, stigma and discrimination that frequently result in harmful practices against persons with mental illness. Previous explorations of the required response to these challenges have identified the need for strong leadership and consistent advocacy as potential drivers of the desired change. The Mental Health Leadership and Advocacy Program (mhLAP) is a project that aims to provide and enhance the acquisition of skills in mental health leadership, service development, advocacy and policy planning and to build partnerships for action. Launched in 2010 to serve the Anglophone countries of The Gambia, Ghana, Liberia, Nigeria, Sierra Leone, this paper describes the components of the program, the experience gained since its initiation, and the achievements made during the three years of its implementation. These achievements include: 1) the annual training in mental health leadership and advocacy which has graduated 96 participants from 9 different African countries and 2) the establishment of a broad coalition of service user groups, non-governmental organizations, media practitioners and mental health professionals in each participating country to implement concerted mental health advocacy efforts that are focused on country-specific priorities

## Introduction

The five Anglophone West African countries of The Gambia, Ghana, Liberia, Nigeria and Sierra Leone share similar mental health profiles with each other and with many other low- and middle-income countries, with low policy priority for mental health, poor funding, inadequate human resources for mental health, widespread misconception about mental health issues, stigmatization and human rights abuses [1-5]. Even though evidence exists for a high burden of mental disorders in the countries [6-8] most of those affected receive no treatment. For example, the World Health Organization (WHO) World Mental Health Survey revealed that only one in five of those with common but serious mental disorders in Nigeria had received any treatment in the preceding 12 months [9]. Similar treatment gaps have been reported in other countries in the region [10]. This gap exists despite considerable evidence that, even within the constraints of low level of resources common to countries in the region, cost-effective interventions are available for most mental, neurological and substance use disorders [6,11]. Thus, even if some of the unmet need for services reflects the scarcity of human and material resources, a fundamental problem underlying the gap is the low priority accorded mental health issues by governments in the region, as well as low levels of knowledge about how to use available resources more efficiently [12,13]. An indication of this low priority is that none of the five countries is implementing a government program on mental health service that is guided by a clear policy.

It is now commonly recognized that effective advocacy has to be a central component of efforts directed at generating political will [14] for increased attention to mental health service improvement and scale-up as well as reducing the level of public stigmatization of the mentally ill [15-19]. Such advocacy presents access to mental health services as a fundamental human right [20,21] and derives its strength from harnessing the commitment and energy of key stakeholders, including mental health professionals, policy makers, service users and families. However, such advocacy requires specific and appropriate skills in order for it to marshal and present arguments that are convincing and actionable. The lack of such skills and the lack of a broad public health perspective among leaders in the mental health community have been identified as the major obstacles to mental health service development in low- and middle-income countries [1,22].

Several important contributions to mental health policy and services development have either been recently implemented or are currently ongoing in sub-Saharan Africa. An example is the Mental Health and Poverty Project (MHaPP), which involved the countries of Ghana, Uganda, Zambia and South Africa [23]. Ghana was the only West African country involved in that project and has made significant progress in the areas of mental health policy and legislation [24]. Sierra Leone and Liberia have also witnessed efforts to develop mental health capacity in the aftermath of civil wars, with varying degrees of success, often provided for short periods of time by international mental health organizations [25].

However, a concerted effort to develop and harness indigenous capacity for sustained advocacy to improve mental health services in this region has been lacking. Furthermore, service user groups and non-governmental organizations advocating for mental health services are few, and the efforts of those that do exist are hampered by insufficient opportunities for capacity building [26]. While the existing mental health policies in the participating countries recommend the integration of mental health care services into primary health care, this has been poorly implemented across the region [27,28]. Mental health is also not captured in the existing health management information systems in these countries.

It is therefore important that opportunities for empowering stakeholders to promote and sustain mental health advocacy campaigns in sub-Saharan Africa, including West Africa, are made available. Such opportunities will align with activities directed at mental health system strengthening, including its integration into general healthcare programs in developing countries [29,30].

## The Mental Health Leadership and Advocacy Program (mhLAP)

The Mental Health Leadership and Advocacy Program (mhLAP) was developed by the Department of Psychiatry, University of Ibadan, Nigeria, in partnership with CBM International, and the Centre for International Health of the University of Melbourne, Australia. Funded by the Australian Aid Agency and CBM, it has two main overlapping goals: 1) capacity building for mental health leadership and advocacy; and 2) development of stakeholder groups with the ability to identify and pursue country-specific mental health service development needs and targets. The aim therefore, is to provide both a top-down, and bottom-up approach to advocacy in countries, which is based on experience of the need for consistent messages from different actors in order to present strong and effectively communicated arguments for change. The program is implemented in the five Anglophone West African countries of The Gambia, Ghana, Liberia, Nigeria and Sierra Leone.

### Program implementation and structure

The mhLAP was launched in 2010, under the overall leadership of the University of Ibadan. The plan was to set up an office in each of the five participating countries, each headed by a Country Facilitator employed full time and working under the day-to-day supervision of a local partner, but ultimately reporting to the Coordinating Office in Ibadan. However, in order to reduce problems that might be associated with multi-country project implementation, the offices were established in a stepwise manner, with the Nigeria office established in the first year, and then joined by Ghana in the second and the other three countries in the third year. This phased commencement of the program had the advantage of ensuring that activities started early in the countries with the largest population and most challenging terrain, while allowing for lessons learned from the initial process to be incorporated into subsequent countries’ activities. It also ensured that the work was incremental and therefore not overwhelming for effective co-ordination and monitoring.

The planning of the programme implementation involved several engagement activities. These activities were led by the Project Director (OG) who visited each of the participating countries to solicit and develop specific partnerships to facilitate the take-off and subsequent implementation of the project. These activities included meetings with the officials of the Mental Health Society of Ghana (MEHSOG), the Executive Director of Basic Needs (Ghana), officials of City of Rest and of Enabling Access (another CBM-led project) in Sierra Leone, the Gambian Federation of the Disabled, the WHO Country Representative in the Gambia, and senior officials of the Ministries of Health of both Liberia and the Gambia (including the Minister of Health of the latter). These engagement activities led to the development of partnerships with individuals and organizations for the specific purpose of providing country-level supervision and oversight for the Country Facilitators and provision of office spaces.

**Figure 1:**
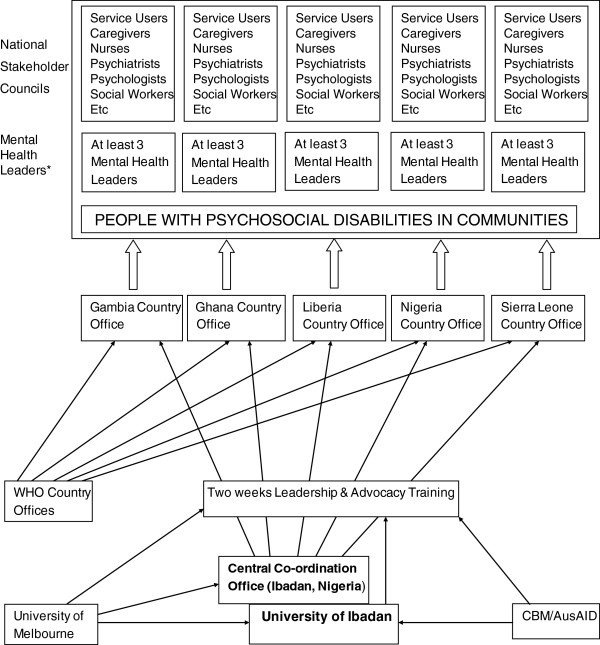
**Organizational structure of MHLAP.** *This includes Ministry of Health officials, and senior Hospital Administrators.

The mhLAP activities are organized and implemented at regional and country levels. See Figure 1 for the organizational structure.

### Training component

The training component of the program is implemented through a two-week annual workshop. Prior to implementation, a curriculum development exercise was organized with the participation of experts in the fields of global mental health, health promotion, communication arts, mental health service evaluation and policy development. An important source of information in the curriculum development was the curriculum of the International Mental Health Leadership Program [31] of the Centre for International Mental Health of the University of Melbourne, with experience of other leadership courses also taken into consideration. The curriculum was informed by current best evidence in the field of public health and health system development. While a major influence in the development of the curriculum was to meet the needs of students from Anglophone West African countries, many of the realities in these countries are very similar to those of much of sub-Saharan Africa, and the content has also taken account of the wider situation in many low- and middle-income countries. Topics covered include: burden of mental, neurological and substance use disorders; organization of mental health services; evaluation of services; mental health financing; mental health policy and legislation; social determinants of mental health; principles and practice of health promotion; the art of communication; stigma of mental illness; and mental health system reform and strengthening. The course also aims to inform the trainees about the strong reciprocal links that exist, at both personal and national levels, between poverty, social determinants and mental health. The expectation is that, at the end of the training, participants will be able to serve as advocates for an approach to responding to the burden of mental disorders within an integrated and evidence-based service for chronic health conditions.

The training course is targeted at mental health leaders, senior and mid-level government officials, policy makers, leaders of civil society, and user and caregiver organizations. The objective is to create a critical mass of informed and empowered opinion leaders who can generate action for positive change in their communities. The structure is interactive, with didactic lectures followed by group discussions and presentations. On the last day of the two-week course, participants are encouraged to prepare and deliver presentations on their proposed plan of action for mental health service development and advocacy upon their return to their respective countries. This provides an avenue for constructive feedback and contributions from other trainees and the faculty to help fine-tune these proposals.

As well as local experts, the faculty for the training has included international experts in global mental health with practical experience of working in low- and middle-income countries. Thus, a multi-disciplinary team of experts from areas such as mental health, public health, language and communication arts, health economics and health promotion has been involved in the training. Non-governmental organizations (NGOs) that have been active in the area of mental health in West Africa, such as CBM (Nigeria, Sierra Leone and Togo) and Basic Needs (Ghana) have also been involved in the training workshops (as well as in program consultative meetings) to share experiences and strengthen collaborative networks.

The training course started in 2010 and has taken place annually since then. The profile of participants from several African countries till date is presented in Table 1.

**Table 1:** Number of participants at the Leadership and Advocacy Course from various African countries from 2010 – 2013

**Country**	**2010**	**2011**	**2012**	**2013**
Nigeria	27	16	10	14
Namibia	3	0	0	0
Ghana	0	4	0	3
Sierra Leone	0	0	8	3
Liberia	0	0	4	3
Gambia	0	0	4	2
Niger	0	0	1	0
South Africa	0	0	0	2
Kenya	0	0	0	2
**TOTAL**	**30**	**20**	**27**	**29**

### Stakeholders councils and country-specific activities

Country offices were established in Nigeria and Ghana, in 2010 and 2011 respectively, while those in Sierra Leone, Liberia and The Gambia were established in 2012. Each office is headed by a full-time Country Facilitator and is housed in a space provided by a country partner. Partners include a mental health user organization, a faith-based non-governmental organization providing care for persons with severe mental disorder, and a WHO country office. The duties of the Country Facilitator include strengthening of links between people involved in mental health in the country, and the implementation of the program of activities guided by these stakeholders. Depending on the local networks of stakeholders, the Facilitators either worked to strengthen existing groups (for example in Ghana), or constituted a National Stakeholders Council (NSC) where no group existed with a broad membership (the Council’s name varies in each country). The aim was to build on local strengths rather than to replicate or create parallel or competing institutions. The reality was that in most countries, while some interest groups existed (professional, traditional, or service user/carer), there was rarely a group aiming to bring all of these diverse interests together.

One of the first activities for the Facilitator in each country was the collection of data to inform a comprehensive situation analysis of the status of mental health services at the onset of the programs. This has been published separately and, as well as guiding decisions about advocacy priorities, will be important as a baseline for subsequent program evaluation.

In each of the participating countries, the NSC is composed of mental health professionals, representatives of user or caregiver organizations, officials of relevant government departments, leaders of non-governmental organizations with interest in mental health or human rights issues, and media practitioners. The NSC in each country serves as an active lobby group to develop and conduct mental health advocacy activities.

The NSC meets at least two times per year to draw up program of activities reflecting identified needs. For example, the NSC may decide that advocacy for a mental health policy would be its main area of action or that it would seek to organize workshops for journalists to discourage them from using derogatory terms when reporting on persons with mental illness. Once high priority activities are decided as the focus of the Council’s attention, the Country Facilitator is given the responsibility of coordinating the activities and reporting back to the Council on the progress of implementation. In carrying out these functions, the Country Facilitator reports to the Project Office in Ibadan from where regular supervisory visits to the country offices are carried out.

The NSC includes graduates of the leadership and advocacy training course in Ibadan, Nigeria. The Country Facilitators, as well as these graduates, are guided by priorities set by a wide coalition of stakeholders in the country, creating a unified message, with the legitimacy that comes from such a broad-based constituency. Indeed, the expectation from the leadership course participants is that they become informed advocates for mental health service development in their respective countries. Thus, at every meeting of the NSC, further in-house capacity building is promoted by having one or two members who have attended the Ibadan training speak on selected topics as a way of informing others. In this way, members of the NSC are provided with the knowledge to enable them to inform the national agenda with respect to mental health, in a systematic and evidence-based manner.

An annual three-day workshop is also organized in Ibadan for selected representative of each country NSC. The workshop provides participants with a forum to discuss and share experience, learn from others, and receive feedback on planned country activities. The workshop also serves as an opportunity for the Project Coordinating Office in Ibadan to receive annual country reports of activities that highlight achievements, challenges and limitations in each of the participating countries.

A summary of the country-specific activities conducted as at the end of 2012 is presented in Table 2.

**Table 2:** Summary of selected country-specific mhLAP activities in Anglophone West African Countries till date

	**Gambia**	**Ghana**	**Liberia**	**Nigeria**	**Sierra Leone**
**Process Indicators**					
Average number of engagement meetings held with policy makers per year	9	3	14	9	14
Number of policy makers and planners participating in each country’s Stakeholders Council	20	3	4	16	5
**Output Indicators**					
Number of stakeholder groups involved in the Stakeholders’ Council	18	14	5	16	35
Number of members who have attended the 2 weeks leadership training course in Ibadan, Nigeria	6	7	7	67	11
Number of advocacy activity by type	TV = 8	TV and radio = 8	Radio = 5	TV = 23	TV and radio = 16
	Radio = 11	SGM* = 5	School outreach = 6	Radio = 28	SGM* = 14
	Newspapers = 19	Workshops = 1		Newspapers = 18	Workshops = 2
	SGM* = 7	Policy meetings = 2	TOTAL = 11	SGM* = 3	Training seminar = 1
	Workshops = 5	TOTAL = 16		Mental Health Walk and Rally =1	AGM^#^= 1
	TOTAL = 50			Printing and distribution of information leaflets	Conference = 1
				Weekly mental health community outreach	TOTAL = 35
				Family support group meeting	
				Prison outreach	
				TOTAL = 73	
Number of engagement meetings with senior Government officials	Minister of Health = 2	Minister of Health = 1	Director of Social welfare	Mental Health Desk Officer, Federal	Minister of Health = 7
	Director of Health = 2	Minister of Justice = 1	Probation officer, Min of Justice	Ministry of Health = 2	Minister of social Welfare, Gender and
	Dir, Health promotion = 3	Minister for Gender, Children	Director of Mental health unit	State Ministry of health (Lagos) = 3	Children’s Affairs = 7
	Director, Social Welfare = 1	and Social Protection = 1	Ministry of health and social welfare	State Ministry of Health (Kano) = 2	TOTAL = 14
	Nat. Assembly = 1	TOTAL = 3	TOTAL = 8	TOTAL = 7	
	TOTAL = 9				
**Outcome Indicators**					
Country-specific situational analysis report prepared	Yes	Yes	Yes	Yes	Yes
Evidence of heightened awareness about the salience of improving mental health services and/or reduction in stigmatization	Plans to review the mental health legislation are ongoing	Self help groups now undertake community mobilization and advocacy programs for mental health.	Efforts to rehabilitate the Catrine Mills rehabilitation centre.	Rehabilitation of homeless mentally ill citizens in some states. Revised mental health policy and legislation are under review	Mental health is now included in the Government’s 5 year vision plan (2012 – 2017).
		Stakeholders strong participants in process of passing Mental Health Act, and now in advocating for its implementation	Mental Health Policy completed		Increased number of civil society groups and NGOs are now interested in mental health
			Draft of mental health legislation under review		Success of annual conference, which has a strong international attendance each year
			New NGOs now focus on Mental health.		Major celebration of World Mental Health Day each year

SGM* = Special Group Meeting.

AGM^#^ = Annual General Meeting.

### Salient achievements

The mhLAP demonstrates a pragmatic and context-specific model for developing capacity for mental health leadership and advocacy across several countries. It is efficient and cost-effective, as it uses the expertise and resources of a centre of excellence in West Africa and global mental health experts while building on the capacities and local knowledge of people with a commitment to promoting change in their own contexts. The emphasis of the program on the importance of capacity building for key stakeholders and policy makers has paid dividends as all the country offices and their activities in the five member countries enjoy official government recognition and cooperation to varying degrees. Furthermore, the partnership with the WHO in some of the participating countries has also ensured that the country offices are able to benefit from the technical assistance and credibility of the WHO. The principle of using a situation analysis to understand local resources and systems before working to strengthen them avoids the risk of doing harm by creating conflict, or wasting resources by creating parallel structures.

The implementation of the mhLAP activities in a step-wise manner in the five participating countries has also been helpful, as it allowed efforts and available expertise to be concentrated initially on only one country in the first year. The lessons and experiences gained were essential in facilitating subsequent efforts to establish the activities in the other member countries. This approach has led to the smooth launching of country offices, each of which has successfully completed a mental health situational analysis. This analysis has helped to focus attention on identifying the salient and specific challenges to be tackled in each country as well as country-specific opportunities for change and development.

In each country, the NSC is evolving into a strong, coherent and authoritative lobby group for mental health policy and service development. The successes of some, for example in Ghana and Sierra Leone, have been documented elsewhere [10,32]. Many of the Councils have been able to effectively co-ordinate with other non-governmental organizations, faith-based and service-user organizations in order to amplify the magnitude of their impact. Reciprocally, selected members of other collaborating groups in the member countries have also been nominated for participation in the leadership training course in Ibadan, further enlarging the group of informed leaders and advocates.

The mhLAP model is sustainable over time, because a growing number of the participants of the leadership and advocacy training course pay to attend the training and many others are sponsored by other institutions, programs or projects for the training. Table 1 shows that an increasing number of participants come from outside the West Africa sub-region, suggesting the relevance of the program beyond West Africa. Furthermore, the commitment and enthusiasm of the NSC members is such that NSC activities are self-funded. In at least one of the participating countries, the Council has transformed itself into a registered non-governmental organization, thus making it possible for it to seek funding and other support for its activities. In Sierra Leone, program funds supported a conference in the first year, but it was such a success that the country’s NSC has been able to independently organize a conference on three further occasions that has grown every year.

In addition to the number and diversity of members who have received training from the participating countries, the impact of the mhLAP activities has also contributed directly to the official recognition of the burden of mental health conditions in the participating countries. In Ghana, Liberia, the Gambia and Sierra Leone, the Stakeholders Councils are focused on supporting the governments to implement existing mental health policy (and legislation in Ghana) or review obsolete mental health legislation. The emphasis of mhLAP on policy and legislation development is strategic and aims to provide a positive framework for achieving the desired changes in mental health services, improve psychosocial rehabilitation and reduce widespread discrimination and stigma. In some countries, for example Nigeria, Ghana and Sierra Leone, the importance of the Councils as an important voice has led to their being invited for consultation on reviews of policy and legislation as well as in planning for service development.

### Challenges and limitations

The West African region is large and diverse. Nigeria alone has an estimated 250 plus distinct ethnic groups, across an extensive terrain that is challenging to cover adequately. Such communication and logistical challenges are multiplied across several countries. It has therefore been difficult to co-ordinate activities across the different countries, but the effective use of the internet (for example sharing resources via email, and holding Skype meetings) has helped in overcoming these problems.

Policy makers and government officials have been slow to respond to calls for reform and change, but this is always a challenging factor in implementing interventions relying on Government processes to bring about change. Even though a revised mental health policy has finally been adopted in Nigeria in 2013 after a long bureaucratic delay, political action on a draft mental health bill is still being awaited. Despite this, the overall response has been positive, albeit at a slower pace than would have been desired. Despite the difficulties in measuring change and attribution to advocacy, the overall picture in the five member countries is still one of positive but slow change.

## Conclusion

The mhLAP is designed to empower patients, caregivers, mental health professionals and policy makers to identify and implement a mental health advocacy agenda that reflects their experience and the specific context of their country. In almost all of the countries involved, this was the first time that such stakeholders have had an opportunity to inform the national agenda with respect to mental health in a systematic and evidence-based manner. The program design and implementation and strategy are derived largely from the cumulative experience of working to improve mental health systems in low and middle income countries of the Department of Psychiatry, University of Ibadan, a WHO Collaborating Centre for Research and Training in Mental Health, Neurosciences and Substance Abuse, complemented by the extensive community experience of CBM in several African countries and that of the Centre for International Mental Health of the University of Melbourne. The most important measure of success though, will be the capacity of the program to translate such principles into practical change in countries through increased motivation, imagination, and strengthened knowledge and skills, of those who have participated in the program. The achievements to date attest to the potential for such a program as a strategy for bringing about change in low- and middle-income countries where policy neglect and lack of resource allocation are a major obstacle to mental health system reform and service scale-up. Those achievements also suggest that practical action can be taken to motivate low- and middle-income countries to channel their efforts into the development of innovative mental health programs that seek to provide evidence-based services, even within the constraints of limited resources. These achievements attest to the power of partnerships. The specific partnerships developed with country-level players such as Basic Needs in Ghana, City of Rest in Sierra Leone as well as with other influential leaders and individuals in the participating countries have been crucial in making the program a regional vehicle for mental health development that is still very much in motion.

## Competing interests

The authors declare no conflict of interest.

## Authors’ contributions

OG conceived the idea and all the authors subsequently contributed to the development, drafting and revision of the article. The first draft was prepared by JA and OG. All the authors approved of the final version.
